# A Review on The Protective Effects of Metformin in Sepsis-Induced Organ Failure

**DOI:** 10.22074/cellj.2020.6286

**Published:** 2019-07-31

**Authors:** Fatima Ismail Hassan, Tina Didari, Fazlullah Khan, Kamal Niaz, Mojtaba Mojtahedzadeh, Mohammad Abdollahi

**Affiliations:** 1. The Institute of Pharmaceutical Sciences (TIPS), Tehran University of Medical Sciences, Tehran, Iran; 2. Department of Toxicology and Pharmacology, Tehran University of Medical Sciences, Tehran, Iran; 3. Department of Clinical Pharmacy, Tehran University of Medical Sciences, Tehran, Iran

**Keywords:** Adenosine Monophosphate-Activated Protein Kinase, Metformin, Multi-Organ Failure, Oxidative Stress, Sepsis

## Abstract

Despite advances in sepsis management, it remains a major intensive-care-unit (ICU) concern. From new prospective, positive effects of metformin, such as anti-oxidant and anti-inflammatory properties are considered potentially beneficial properties for management of septic patients. This article reviewed the potential ameliorative effects of metformin in sepsis-induced organ failure. Information were retrieved from PubMed, Scopus, Embase, and Google Scholar. Multi-organ damage, oxidative stress, inflammatory cytokine stimulation, and altered circulation are hallmarks of sepsis. Metformin exerts its effect via adenosine monophosphate-activated protein kinase (AMPK) activation. It improves sepsis-induced organ failure by inhibiting the production of reactive oxygen species (ROS) and pro-inflammatory cytokines, preventing the activation of transcription factors related to inflammation, decreasing neutrophil accumulation/infiltration, and also maintaining mitochondrial membrane potential. Studies reported the safety of metformin therapeutic doses, with no evidence of lactic acidosis, in septic patients.

## Introduction

Recently, at the 45^th^ Congress of Society of Critical Care Medicine in Florida, sepsis was defined as a life- threatening condition associated with organ damageas a result of dysregulated host immune response (1). Septic shock has been described as persistent elevationin lactate levels above 2 mmol/L despite adequate fluidresuscitation and elevation in mean arterial pressure to over 65 mmHg that requires administration of a vasopressor (2). Recent evidence suggests that people at risk of developing sepsis, are usually critically ill, elderly, and immunocompromised patients (3). There are other acute and chronic conditions that can cause systemic inflammatory response and events similar to those observed in sepsis, including burst of inflammatory mediators, hemorrhagic shock, pancreatitis, trauma, and ischemic conditions (4). To date, management of sepsis has been challenged due to its complex nature. Experimental studies have proven the possible alleviative effect of anti- inflammatory drugs in sepsis. The protective effects of metformin mediated by anti-oxidative and anti- inflammatory mechanisms, were demonstrated in recent studies (5-7). Such evidence made metformin a potential candidate for sepsis management. Lactic acidosis has emerged as the most concerning issue in metformin users, since it affects lactate clearance and metabolism (8). However, some studies abrogated of the association between therapeutic doses of metformin and lactic acidosis occurrence. Studies on metformin reported an improvement in sepsis-induced inflammatory and oxidative stress conditions, as well as improved mortality at different tested doses and in various models (9, 10). This article aimed at reviewing the ameliorative effects of metformin in sepsis-related organ failure and discussing the underlying molecular mechanisms. 

### Overview of sepsis 

Sepsis is a lethal situation with a high mortality rate in intensive-care-unit (ICU) hospitalized persons (11). It is characterized by severe blood or tissue infection with a burst of inflammatory markers and subsequent mitochondrial dysfunction due to the presence of molecules released by microbes or stressed cells. Molecules released from these microbes stimulate inflammatory responses, which in turn lead to tissue damage and production of some mediators that can exacerbate inflammation and tissue damage, and eventually cause organ failure (12). These include inflammatory mediators such as high mobility box protein (HMGB1) which can serve as a classic damage-associated molecular pattern (DAMP) molecule in the late phase of sepsis. Sepsis is associated with risk factors such as age, delayed treatment, persistent infection, immune suppression, and previous illness (13). Polymorphism of genes encoding inflammatory mediators, heat shock proteins, and toll-like receptors (TLRs), can influence the incidence and severity of sepsis (14). 

**Fig.1:**
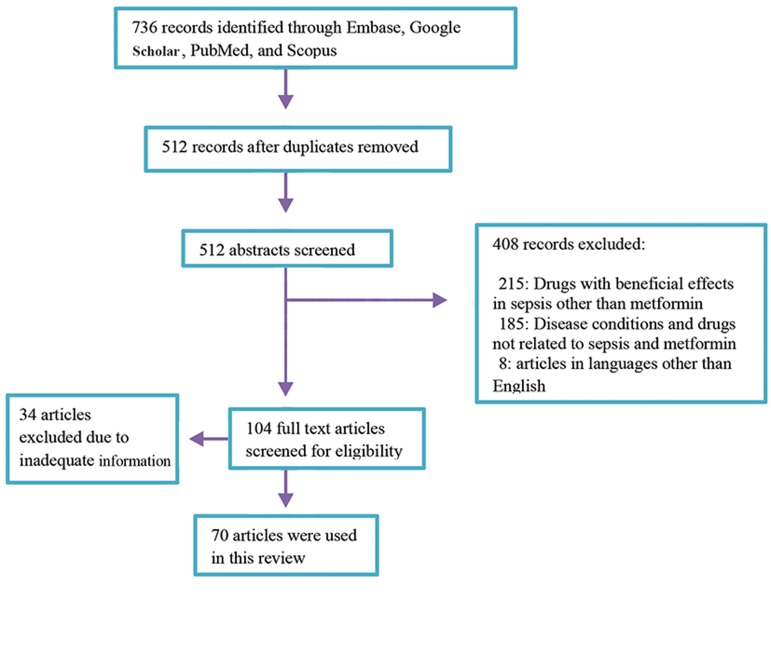
Search strategy indicating inclusion and exclusion criteria.

### Biochemical events in sepsis 

**Fig.2:**
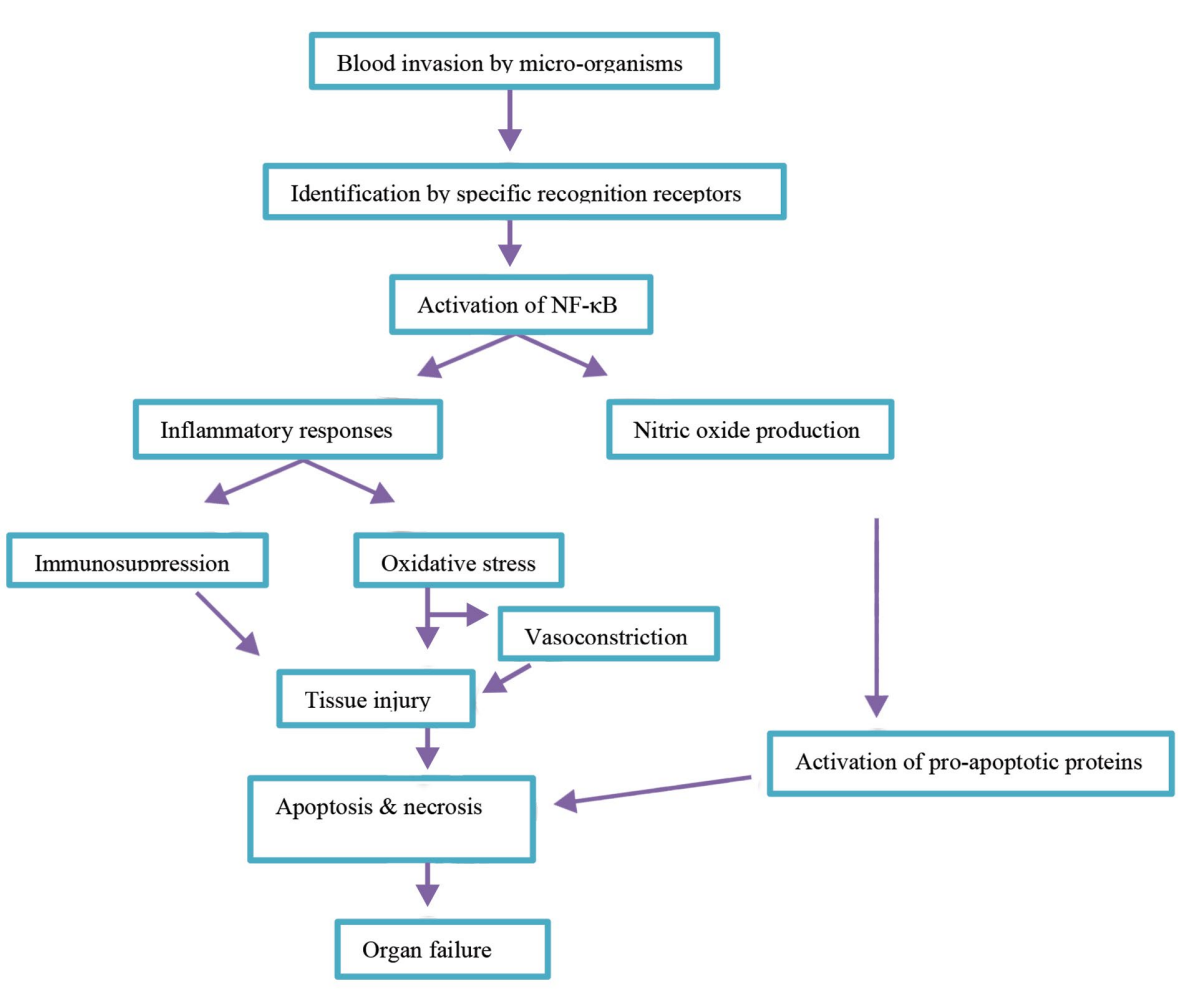
A summary of biochemical events occurring during sepsis.

Sepsis involves the release of molecules exclusively synthesized by microbes [lipopolysaccharides (LPS), peptidoglycan, and bacterial lipoproteins] recognized by pattern recognition receptors (CD14, TLRs) which send warning signals to the host, and induce stimulation of cytokines, chemokines, and complement system, and subsequent activation of the immune system cells neutrophils, monocytes, and macrophages (1517). It involves both cellular and molecular events which occur as a result of stimulation of inflammatory mediators, oxidative stress markers, apoptosis, as well as interference with some neurotransmitters and signaling pathways (Fig.1). These events lead to tissue injury and subsequent organ damage/failure. Sepsis is associated with increased production of inflammatory cytokines in response to the entrance of microorganisms into the blood (18). Sepsis is comprised of two inflammatory stages namely, an acute phase and subsequently, a late-phase characterized by a systemic inflammatory response syndrome (SIRS) and a complimentary anti-inflammatory response syndrome (CARS), respectively (19). The typical characteristic of these stages are exaggerated productions of pro- and anti-inflammatory cytokines or chemokines which lead to a cytokine storm. These cytokines exhibit both beneficial and deleterious effects as although they are produced to eliminate infection, their overproduction/overactivity can lead to immunosuppression, tissue damage, and organ failure (19, 20). Proinflammatory cytokines stimulate systemic responses while anti-inflammatory cytokines inhibit such responses and initiate wound healing (19). These early response regulators (i.e. proinflammatory cytokines) are implicated in SIRS, while the anti- inflammatory mediators secreted during CARS play immunoregulatory roles in sepsis (20, 21). These hyper-inflammatory responses affect the balance between endogenous oxidants and antioxidants and cause immunosuppression which leads to tissue injury, cell death, and organ failure. Excessive activation of NF-κB stimulated the production of reactive oxygen species (ROS) and affected endogenous antioxidant activity, which in turn caused lung damage and distal organs apoptosis in septic mice (22). Redox imbalance due to production of ROS can affect respiratory chain activities and mitochondrial structure (23). These events increase oxidative stress, tissue injury, and organ failure by affecting blood flow of various organs. Increased production of ROS leads to vasoconstriction and subsequent decreased blood flow of vital organs (24). Another important parameter in sepsis is accumulation of lactate which is associated with inactivation of pyruvate dehydrogenase (PDH) enzyme. Tumor necrosis factor (TNF) may be involved in preventing PDH activity in the skeletal muscles of rats with chronic abdominal sepsis (25). Blood or serum lactate concentration during sepsis is regarded as the most important indicator of morbidity and mortality during sepsis. Hyperlactatemia was shown to be associated with sepsis severity. In this regard, increment of lactate clearance was found to be associated with improved clinical outcomes and decreased mortality probably by resolving hypoxia. Several clinical trials reported the improved clinical outcomes in patients with sepsis and septic shock, following adequate lactate clearance. A 10% increase in lactate clearance was associated with one-score decrease in acute physiology and chronic health evaluation II (APACHE II), and an approximately 11% decrease in mortality among the septic patients (26). Decreased APACHE II score, days spent in the ICU, and 28-day mortality in groups with 10-30% lactate clearances were also observed in patients with severe sepsis and septic shock (27). Recent findings using *Escherichia coli*-infected cells (*in vitro*), and LPS and cecal ligation and puncture (CLP)-induced septic mice (*in vivo*) revealed the role of adenosine monophosphate kinase (AMPK) pathway in sepsis severity as suppression of this pathway led to an increase in pyruvate kinase isozyme M2 (PKM2)dependent aerobic glycolysis. This increases HMGB1 release, lactate accumulation, and mortality (28). Activation of NF-κB pathway that involves nitric oxide (NO) production was shown to be associated with activation of pro-apoptotic proteins signaling such as the Fas and Fasl which led to apoptosis in A549 human lung epithelial cells and mice treated with LPS (29). Common molecular events that occur during sepsis are inflammation, oxidative stress, and apoptosis which lead to the observed deleterious effects. Hence, agents that have the ability to block these effects, may be beneficial in sepsis management. Studies reported that the antioxidant, anti-inflammatory, and anti-apoptotic effects of metformin are mediated by blockade or enhancement of the involved molecular pathways; thus, it may affect sepsis via same mechanisms (Fig.2). 

### Metformin 

Metformin has several functions in addition to its anti hyperglycemic effects. Inhibition of respiratory chain complex in the mitochondria is a primary action of metformin (30). It exerts its effect by inhibiting complex I of the electron transport chain (ETC) which increases AMP/ATP ratio and subsequently induces AMPK activation (31). This effect was observed in perfused liver, skeletal muscles, endothelial cells, pancreatic beta cells, and neurons (32-34). AMPK plays significant roles in cellular responses to danger signals (35). 

The protective effect of metformin is majorly mediated through activation of AMPK and inhibition of NF-κB pathway (36). It decreases mitochondrial respiration which renders cells less energetic, increases aerobic glycolysis, and reduces glucose metabolism through the citric cycle. Metformin reduces tumor cell growth by preventing lipid membrane biosynthesis in the mitochondria (37). It was shown that inhibition of complex I of ETC in cancer cells prevented oxidative phosphorylation, which in turn, decreased cell multiplication both *in vitro* and *in vivo*. Metformin lessened the activation of apoptotic cascade by inhibiting the permeability transition pore (PTP) opening (38). It also attenuated kidney injury induced by gentamicin in rats (7). 

Co-administration of metformin with garlic also prevented gentamicin-induced kidney injury and suppressed diabetes- induced podocyte loss in diabetic neuropathy (39, 40). Metformin was reported to exert potent antioxidant activity, especially in oxidative stress-induced damage in diabetic patients (5). It reduces cardiovascular-associated mortality and morbidity in both diabetic and non-diabetic patients (6). Metformin was shown to decrease blood pressure, left ventricular mass, and cholesterol, triglycerides, and fibrinogen levels in obese non-diabetic hypertensive women (41). It also decreased blood pressure and hyperandrogenemia in women with polycystic ovarian syndrome (42). Metformin also reduced myocardial infarct size by preventing membrane PTP (mPTP) opening via the phosphatidylinositol-3kinase (PI3K) pathway (43). Metformin prevented brain mitochondrial dysfunction by decreasing oxidative stress levels and reducing insulin resistance (44). A combination of metformin and insulin given to critically ill patients reduced the incidence of insulin resistance, adverse effects associated with high-dose insulin therapy, as well as inflammatory cytokines production (45-47). 

Other animal studies reported metformin’s ability to promote neurogenesis, enhance spatial memory, protect against cerebral ischemia, and stimulate angiogenesis (48, 49). Sepsis includes similar biochemical events; hence, metformin may protect against sepsis-induced organ injury via above-noted mechanisms. Some studies reported beneficial effects of metformin on sepsis. Despite these promising effects, metformin is still contraindicated in organ failure due to risk of lactic acidosis. However, some studies reported the safety of metformin at therapeutic doses with little or no incidence of lactic acidosis but improvement of morbidity and mortality. 

### The safety of metformin in sepsis

Metformin was shown to cause lactate accumulation by inhibiting or reducing lactate metabolism and clearance in the liver through induction of hypoxia or excessive inhibition of mitochondrial respiration (50). Metformin was also found to be associated with non-hypoxic lactic acidosis with an increased mortality rate of about 50%. This is the main reason for metformin contraindication in critically ill patients, especially in sepsis. However, this effect of metformin on mitochondrial respiration that causes lactate accumulation was reported at therapeutic doses. Some studies reported metformin-associated lactic acidosis (MALA) only in case of overdose which is rare, while some studies found no MALA even at very high doses of the drug. MALA in sepsis may not be significant as the benefit outweighs the risk and the usual doses are within the therapeutic limit. The following studies reported little or no aggravating effects for metformin in terms of sepsis-related mortality. Metformin did not affect survival rate in sepsis at high doses (500 mg/kg) in mice, which was similar to smaller doses (50 mg/kg) used in clinical practice (9). In another study, patients with suspected sepsis were recruited from the emergency department (ED); higher lactate levels and incidence of hyperlactatemia were more common in metformin users compared to non-users though it did not indicate poor prognosis (51). A clinical trial reported lower incidence of hospital mortality in metformin users compared to non-users, while lactate, bicarbonate, and blood pH levels were similar between the two groups (10). This inferred that metformin did not increase the lactate level, but improved mortality. Another study similarly reported that metformin had no significant impact on lactate levels, clearance, and normalization, over a 24-hour period in patients with severe sepsis and septic shock (52). These studies suggest that though high lactate concentration indicates poor prognosis in sepsis, metformin use may not increase lactate levels, which is proportional to disease severity or increase in mortality rate. Further studies on the effect of metformin on lactate are needed to clarify MALA incidence at therapeutic doses. 

### The effects of metformin on sepsis-induced organ failure 

The biochemical events that occur in sepsis usually result in organ failure and death. Studies reported metformin induces beneficial effects against sepsis by interfering with inflammatory markers and other molecular processes, which are mainly mediated via AMPK activation. Metformin was reported to increase bacterial killing by enhancing neutrophil chemotaxis via AMPK activation. It promoted the chemotactic and bacterial killing ability of neutrophils in an LPS-induced model (53). This suggests that AMPK activation by metformin may prevent the influence of molecules released by microbes and their subsequent deleterious events. Some reported protective effects of metformin on sepsis-induced organ failure are presented in Table 1. 

**Table 1:** Protective effects of metformin on sepsis


Organ	Model	Mechanism	References

Brain	CLP	Inhibition of oxidative stress and apoptosis, and increased BBB integrity.	(54)
Heart	LPS	Suppression of TLRs, and inhibition of MPO activity and inflammatory responses.	(55-58)
Lungs	LPS, CLP	Inhibition of inflammatory cytokines, activation of ATF-3, and enhance neutrophil chemotaxis, inhibition of neutrophil and macrophage infiltration, and suppression of TLR signaling.	(59-63)
Liver	LPS	Inhibition of pro-inflammatory cytokine production, decreased the expression of PAI-1 mRNA and PAI-1 protein, decreased MPO activity and tissue asymmetric dimethyarginine levels, and restored glutathione.	(64, 65)


CLP; Cecal ligation and puncture, LPS; Lipopolysaccharides, BBB; Blood brain barrier, TLR; Toll-like receptor, MPO; Myeloperoxidase, ATF; Activating transcription factor, and PAI-1; Plasminogen activator inhibitor type-1.

### The brain 

Metformin in different brain injury models could improve brain function and prevent injury. The effects of metformin on sepsis-induced brain injury were also reported; metformin could enhance blood brain barrier (BBB) integrity and attenuate brain injury (54). Metformin improved brain function by preventing brain injury through increased expression of specialized tight junction proteins (claudins), decreased oxidative stress markers, and attenuated apoptosis. Tight junction proteins regulate the passage of molecules across the BBB; thus, any alteration in their concentration will affect the permeability leading to increased brain injury (54, 66). More studies on involved mechanisms and pathways are needed. 

### The heart

In the heart, the consequence of sepsis is microcirculatory failure, right and left ventricular dysfunction, myocardial infarction, and many other forms of heart failure. Metformin exerts its cardioprotective effects via suppression of TLRs. It preserved left ventricular function by reducing myeloperoxidase (MPO) activity and TNF alpha (TNF-α) level, both in the serum and heart tissue. This effect was observed to be mediated via AMPK activation (55). Metformin also inhibited local immune response in the isolated rat heart through AMPK and TLRs pathways (56). Also, metformin could protect against myocardial dysfunction by increasing the expression of genes related to cardiac metabolism, enhancement of fatty acid oxidation and ATP synthesis, while decreasing glucose transport and inflammatory responses (57). Metformin protected against endotoxin-induced acute myocarditis by inhibiting pro-inflammatory cytokines, suppressing the expression of MPO, and decreasing creatine kinase myocardial band and brain natriuretic peptide (58). 

### The lungs

Metformin can reduce inflammation-induced endotoxemia via inhibition of TNF-α, interleukin 1 beta (IL-1ß), and HMGB1 release as well as suppression of MPO activity (59). Metformin was shown to decrease LPS-induced edema and lung permeability through AMPK activation (35). Its anti- inflammatory effects were reported to be mediated through activating transcription factor (ATF-3) (60). The anti-inflammatory effects of metformin via inhibition of TNF-α activity was studied in an in vitro equine whole blood assay (61). Through induction of AMPK pathway, metformin protected against sepsis and improved survival in diabetic mice by reducing lung permeability and decreasing the expression of pro-inflammatory cytokines (67). AMPK activation by metformin was shown to restore and increase the levels of ETC complexes, diminish accumulation of the immunosuppressive transcriptional factor alpha (HIF1a), reduce cells’ tolerance to endotoxin challenge, and prevent the abnormal neutrophil movement caused by LPS (68). In the same study, metformin also improved innate immune ability to eliminate *Pseudomonas aeruginosa* and restore lung balance by inhibiting pro- inflammatory cytokines in bronchioalveolar lavage (BAL). In another study, metformin decreased cytokine production, BAL protein expression, and decreased lung edema in LPS-treated rats; also, neutrophil and macrophage infiltration and MPO activity were prevented, while AMPK-α1 expression was promoted by metformin (62). Metformin decreased acute lung injury by suppressing TLR-4 signaling in LPS-treated rats (63). This effect was mediated via activation of AMPK which reduced LPS-induced expression of TLR4, levels of myeloid differentiation primary response protein 88 (MyD88), NF-κB, and TNFa. It also up-regulated the p-AMPKα/AMPKα ratio by 22% and reduced the congestion and inflammatory cells infiltration into the alveolar walls. It also decreased MPO activity by 37%. Recently, the anti-inflammatory and anti-oxidative effects of metformin on sepsis- induced lung injury were reported (69). 

### The liver 

Liver injury due to sepsis was shown to be induced via oxidative damage, inflammatory response, and neutrophil infiltration. Metformin decreased the expression of inflammatory biomarkers and thrombosis by reducing the expression of hepatic plasminogen activator inhibitor type-1 (PAI-1) mRNA and plasma PAI-1 protein, which was related to the inhibition of hepatic urokinase plasminogen activator activity and an increase in fibrin deposition in a rat model of endotoxic shock (64). Metformin prevented LPS-induced liver injury by reducing MPO activity and asymmetric dimethyarginine tissue level, as well as restoring the activity of antioxidants such as glutathione (65).

### The kidney 

Activation of the innate immune system can cause renal failure by stimulating the secretion of pro-inflammatory mediators which trigger the release of toxic oxygen radicals, protease, and cytokines, that in turn lead to increased vascular permeability, capillary leakage, and impaired oxygen extraction, with subsequent hypoperfusion and hypoxia (70). 

The beneficial effects of metformin on sepsis- induced kidney injury were not reported. However, the importance of oxidative stress and inflammation in the pathophysiology of kidney injury/failure indicates the potential role of metformin in management of such conditions. The beneficial effects reported in other models may support this claim. Studies on the effect of metformin on sepsis-induced kidney injury are required.

## Conclusion

Sepsis leads to multi-organ damage via inflammation and oxidative stress induced by production of pro- inflammatory cytokines and ROS. This happens through NF-κB and other signaling pathways. Metformin, through AMPK activation, suppresses NF-κB signaling which leads to inhibition of the production of pro-inflammatory cytokines especially IL-1ß, IL-6, and TNF-α, in different organs. Hence, metformin was shown to improve organ injury and mortality in sepsis via its action on these mediators. The major limitation of metformin therapy may be the induction of lactic acidosis (MALA) which may not be a matter of concern as its occurrence was only reported at high doses of metformin in the presence of other risk factors. Further experimental studies are required to ascertain the impact of metformin on sepsis- induced organ failure and its safety. 
